# Signal-to-noise optimization and evaluation of a home-made visible diode-array spectrophotometer

**DOI:** 10.1155/S146392469300029X

**Published:** 1993

**Authors:** Ivo M. Raimundo, Jr., Celio Pasquini

**Affiliations:** Instituto de Ouímica Universidade Estadual de Campinas CEP 13081.970 Campinas, SP CP 6154 Brazil

## Abstract

This paper describes a simple low-cost multichannel visible spectrophotometer built with an RL512G EGG-Reticon photodiode array. A symmetric Czerny-Turner optical design was employed; instrument control was via a single-board microcomputer based on the 8085 Intel microprocessor. Spectral intensity data are stored in the single-board's RAM and then transferred to an IBM-AT 3865X compatible microcomputer through a RS-232C interface. This external microcomputer processes the data to recover transmittance, absorbance or relative intensity of the spectra. The signal-to-noise ratio and dynamic range were improved by using variable integration times, which increase during the same scan; and by the use of either weighted or unweighted sliding average of consecutive diodes. The instrument is suitable for automatic methods requiring quasi-simultaneous multiwavelength detections, such as multivariative calibration and flow-injection gradient scan techniques.


Journal of Automatic Chemistry, Vol. 15, No. 6 (November-December 1993), pp. 227-232

### Signal-to-noise optimization and evaluation of a home-made visible diode-array spectrophotometer

**Ivo M. Raimundo, Jr. and Celio Pasquini**
*Instituto de Química, Universidade Estadual de Campinas, CP 6154, CEP 13081.970, Campinas, SP, Brazil*

*This paper describes a simple low-cost multichannel visible spectrophotometer built with an RL512G EGG-Reticon photodiode array. A symmetric Czerny-Turner optical design was employed; instrument control was via a single-board microcomputer based on the 8085 Intel microprocessor. Spectral intensity data are stored in the single-board's RAM and then transferred to an IBM-AT 386SX compatible microcomputer through a RS-232C interface. This external microcomputer processes the data to recover transmittance, absorbance or relative intensity of the spectra. The signal-to-noise ratio and dynamic range were improved by using variable integration times, which increase during the same scan; and by the use of either weighted or unweighted sliding average of consecutive diodes. The instrument is suitable for automatic methods requiring quasi-simultaneous multiwavelength detections, such as multivariative calibration and flow-injection gradient scan techniques.*

### Introduction

Multiwavelength spectrophotometers are increasingly being used with new analytical techniques based on continuous-flow analysis [1-4]; and flow methodologies have been developed to allow mixtures to be used without prior separation [3-7]. Therefore, it is necessary to have complete spectral data; in addition, computer-controlled flow instruments mean that it is possible to build sequential and/or simultaneous analysers demanding fast wavelength selection in the monitoring process of different analytes. Previous attempts at automatic wavelength selection in continuous flow instruments have been based in stepper motors coupled to the monochromator; the scan speed is only 10 nm/s [8].

Diode array spectrophotometers are particularly suitable for these applications because they do not contain any moving parts, so they are free of mechanical stress and less prone to failure. The scan speed of the entire array can be achieved in a few milliseconds, which allows easy implementation of flow techniques that require fast wavelength selection or wavelength scan.

There are commercial instruments available but they are expensive and often cannot be justified by smaller laboratories. In addition, commercial instruments include features which are unnecessary for routine work and they are not easily to adapt to a specific application, for example it can be difficult to obtain the information needed to write software.

There have been other attempts to automate diode array spectrophotometers but using recently introduced sensor devices, which are very expensive. Caceci [9] described a simple diode array spectrophotometer based on an Optical Multichannel Analyser (OMA) for the acquisition of spectra of radioactive solutions. Resolution was good (0·12 nm), a spectrum can be produced in 10 ms with inter-spectrum intervals as low as 10 ms, but commercial software was used and the signal/noise ratio (standard deviation of 0·017 AU, after base-line drift compensation) was poor. Lepla and Horlick [10] built an instrument for inductively coupled plasma-atomic emission spectrometry using an Hamamatsu S2304-1024Q PDA and Reticon 1024S array. Their instrument was constructed with high resolution and high signal/noise ratio, and, therefore, it was expensive. Naffrechoux *et al.* [11] described a diode array ultra-violet detector based on Hamamatsu S3901 sensor for continuous monitoring of water quality. Despite the short time (less than 3 s) necessary to obtain one spectrum (acquisition, mathematical treatment and storage), the instrument had a limited dynamic range for wavelengths lower than 225 nm.

This paper describes a computer-controlled lower-cost visible photodiode array spectrophotometer. The sensor array used is the RL512G (EGG-Reticon); the sensor array can be run at 1 MHz and is usually employed in facsimile machines. The sensor array shows spectral response in the visible range of the spectrum.

### Experimental

### *Optical design of the spectrophotometer*

A simple symmetrical Czerny-Turner optical design was employed in the spectrophotometer. The concave mirrors are 50 mm in diameter and 100 mm foci. An adjustable entrace slit has a coupling device to allow optical guides to be used. Light is directed from the entrance slit to a collimator mirror and then to a dispersion grating (Edmond Scientific—600 lines/inch, blazed at 500 nm). The dispersed light is collected by another concave mirror and focused on the photodiode array sensor (EGG-Reticon RL512G). The optical arrangement is such that the first photodiode accessed in a scanning procedure is exposed to the red portion of the spectrum. The diode array was assembled on the back of a printed circuit board containing the basic electronics employed to extract the intensities of the signals. The board was fixed on an optical support to allow adjustment of the sensor array position. All optical parts were fixed in a 10 mm thick aluminum plate.

0142-0453/93 $10.00 © 1993 Taylor & Francis Ltd.

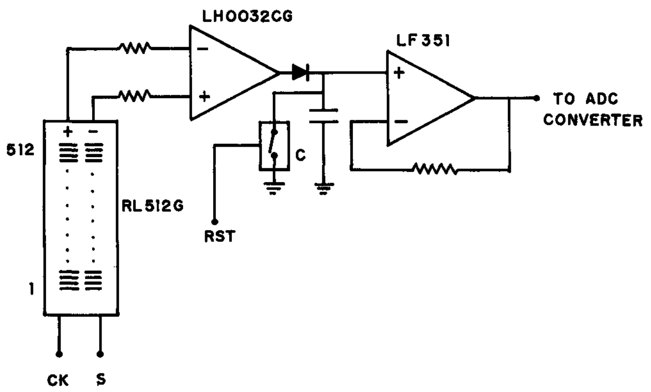

*Figure 1. Schematic diagram of the analogue circuit for signal extraction from the diode array. CK, clock signal; S, start of scan signal; RST, peak-holder reset; C, CMOS analogue switch.*

*Basic analogue electronics*

The photodiode array requires analogue electronics to extract the signal intensities for each wavelength (the circuitry is shown in figure 1). The capacitors associated with each one of the 512 photodiodes are charged after each reading. Thereafter, the photocurrent begins to discharge the capacitors and the amount of current necessary to recharge them is proportional to the light intensity incident on the diode and on the exposure time (integration time). A peak-holder circuit ensures that a static signal is present for the analogue-to-digital conversion. The high-quality LH0032CG operational amplifier converts the current pulse to a voltage pulse. The gain is set to obtain full-scale analogue-to-digital conversion (2·5 V) when the maximum light intensity falls on the array.

*Digital electronics*

The photodiode array sensor is controlled by a single-board SDK 8085 microcomputer based on the 8085 Intel microprocessor running at 3·072 MHz. On the board's expansion area is an 8-bit analogue-to-digital converter (ZN448), a USART (8251A) and 4 kbytes of static RAM (two ICs 6116). An 8155 programmable I/O port reads the ADC and provides the logical signals necessary for data extraction from the diode array. The single-board microcomputer communicates with the main external microcomputer (IBM-AT 386SX compatible, with 2 Mbytes of RAM) through an RS-232C serial interface (implemented on the single-board using the IC 8251A) running at a baud rate of 9600. The 8155 generates three logical control signals directly related to the data extraction from the diode array. The RL512G device requires a negative TTL transition applied to pin 2 to start the scan. Once a scan is started, each clock pulse applied to pin 17 causes the recharging current of each photodiode to be compared with that of a dummy diode in order to minimize noise generated by the action of the internal shift register. A current pulse, which is converted to a voltage pulse by the LH0032CG, is generated by a differential video output. The peak-holder is active at this point and the maximum voltage is stored until the ADC can make its reading. Another logical pulse is then applied to the CMOS analogue switch. The hold capacitor is discharged and another reading cycle can begin. The entire array will have been sequentially read after 512 clock pulses.

*Software for the spectrophotometer*

The first piece of software for the spectrophotometer is an 8085 assembler program which is loaded onto the single-board microcomputer. The micro has a small machine code program which allows it to load the assembler program (this small program was recorded just below the address area in the EPROM reserved for the single-board monitor program). Manipulating software in this way is useful as the machine code necessary to implement the spectrophotometer functions and any of its applications (for example continuous-flow analysis) can be developed on the external microcomputer (using its editing capability and friendly interaction with the user) and then tested in the single-board.

After the machine code has been loaded, the external microcomputer can send commands (in single ASCII characters) to the single-board micro. The commands instruct the single-board to run small machine code routines that perform the control, data acquisition and communication functions. In addition, such parameters as the number of scans, scan speed, and time interval between successive scans, can be transferred to the single-board micro through the serial interface.

The second piece of software used was written in QuickBasic 4·5 to communicate with the single-board microcomputer. Its first task is to load the machine code program onto the single-board RAM and to instruct the 8085 microcomputer to run it. Friendly menus are then displayed which allow the user to obtain spectral data from the single-board of the instrument. Data are transferred from the single-board micro as 512 intensities expressed as binary numbers between 0 to 255. The QuickBasic program is capable of processing the data to display absorbance, transmittance or intensity spectra. For the two first types of spectra, the software will request reference intensities which enable the absorbance or transmittance to be calculated.

*Scanning procedures*

Two scanning procedures were developed to be used for spectral data acquisition. The procedures are contained in small machine code programs loaded by the external microcomputer onto the single-board microcomputer.

The first procedure attempts to scan the sensor array, keeping the same integration time for each diode. The scan time is then equal to the integration time and its minimum value is established by the speed at which the microprocessor executes the machine code instructions necessary to read the whole array. The minimum value for this procedure is 44 ms. Spectral data are extracted from the array by performing a false initial scan to reset all photodiodes to avoid eventual saturation due either to dark current, or to long elapsed integration time interval between two requests for spectral data. This false scan resembles a true scan in all respects in order to maintain the same integration time. Therefore, the first 512 bytes stored in the single-board RAM correspond to a false scan and are not sent to the external microcomputer. Integration time is adjusted by setting the initial value of a counter register, which decreases to zero after each diode reading.

The second procedure was developed to minimize the effects of the different intensities of the spectral signals obtained by the sensors when exposed to light of different wavelengths. The whole array is first scanned very quickly by pulsing, at the maximum frequency allowed by the 8085 CPU, the array clocking signal; no readings are made during this scan. Second, all diode signals are integrated. Finally, a new scan is made and data is acquired. In this valid scan, the first group of 256 photodiodes are scanned at the maximum possible frequency and the resting 256 photodiodes are scanned at a regularly decreasing frequency. This procedure means that all signals are obtained under different integration time intervals which increase when scanning from the first to the last sensor. The minimum scan time interval possible is 300 ms if this procedure is employed.

### *Data processing*

The spectral data acquired from the spectrophotometer need post-acquisition treatment to improve the signal-to-noise ratio.

If more than one scan had been performed, the average of the intensity data was used for subsequent treatment. Two smoothing procedures were investigated: first, an unweighted sliding average smoothing [12] of the signals of three or five photodiodes; secondly, a weighted sliding average [13] of three or five signals.

The absorbance calculation for a photodiode $n$ was made by using the equation:

$$A(n) = \log \frac{(I_0(n) - D(n) + K)}{(I(n) - D(n) + K)}$$

where $I_0(n)$ is the signal intensity of the reference (for example blank solution), $I(n)$ is the signal intensity of the sample solution, $D(n)$ is the dark signal. The K constant accounts to the correction for the intensities at integration time equal to zero. This constant was determined experimentally by scanning the array exposed to dispersed light, using the first procedure. Several spectra were obtained with different integration times. The plot of the intensities versus integration time for any diode gave a straight line, for which extrapolation to an integration time equal to zero intercepted the intensity axis at the same negative value. The absolute value of the intercept was defined as the K constant.

### *Calibration*

The correspondence of the diodes with the spectral wavelengths can be established using a mercury lamp source, or any standard solution such as a neodymium solution, and looking for the localization of emission lines or absorption peaks present in the spectrum. Typical spectra obtained for the calibration procedure are shown in figures 4($a$) and 5($a$). Using the calibration data, the software automatically converts the diode number (1 to 512) to the light wavelength that reaches its surface. In order to calibrate, the user must move a graphic cursor through the spectrum and introduce the known wavelength of its position (usually a peak in the spectrum). At least three wavelengths are necessary to perform a good calibration. The calibration routine will display the linear regression made with the calibration points, and store the parameters necessary to convert the diode number to wavelength units. The calibration parameters are stored in a control file that is accessed each time the program is run for the first time. The file is automatically updated if a new calibration is successfuly completed.

### *Solutions*

Potassium permanganate solutions with concentrations in the $5{\cdot}0 \times 10^{-5}$ to $8{\cdot}0 \times 10^{-4}$ mol.dm$^{-3}$ range were prepared by diluting $1{\cdot}0 \times 10^{-2}$ mol.dm$^{-3}$ of a stock solution. Fresh solutions were always employed. A $5{\cdot}0 \times 10^{-2}$ mol.dm$^{-3}$ neodymium chloride solution was prepared from carbonate salt of analytical grade and hydrochloric acid. A $1{\cdot}0 \times 10^{-3}$ mol.dm$^{-3}$ potassium dichromate solution in $1{\cdot}0 \times 10^{-1}$ mol.dm$^{-3}$ sulphuric acid was prepared from analytical grade salt and concentrated acid.

### Results and discussion

The diode array multichannel spectrophotometer was built for use as a detector in continuous sequential flow analysers. Therefore a flow cell with a 10 mm optical path and 2 mm inner diameter was placed between two 500 mm long, 3 mm diameter glass optical cables. One cable guides the light from the halogen source to the cell, and the other guides the light leaving the cell to the entrance slit of the spectrophotometer. The performance of the instrument was evaluated using this assembly.

The single board can store up to seven spectra in the expanded RAM. However, if the scan is made with the first procedure, then only six scans are available (the first one is false). When these data are transferred to the external microcomputer through the RS-232C serial port, they are averaged and stored in a file named by the user. The time needed to transfer one scan (512 bytes) is about 600 ms; and to average seven spectra and store the result in a file it is about 850 ms. Therefore, spectra can be obtained in intervals as low as 1·5 s. However, averaged spectra have a higher signal-to-noise ratio and spectra were generally obtained after an average of five scans (the time to transfer data, therefore, was 3 s), except where mentioned.

Figure 2 shows an emission spectrum obtained with the first scanning procedure (figure 2[$a$]) and the second procedure (figure 2[$b$]) for a continuous halogen source, at scan time of 63 and 450 ms, respectively.

The software control of the scan rate, and, therefore, of the integration time, is very useful for improving weak signals. Figure 2($a$) demonstrates that the intensity registered by each diode is not constant, but, rather,

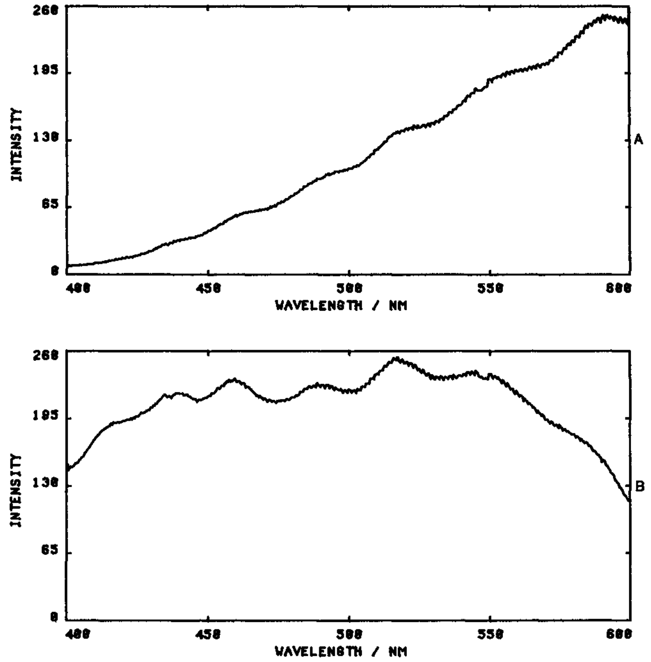

*Figure 2. Emission spectra for a continuous halogen source: (a) using the first scanning procedure (scan time = 63 ms); and (b) using the second scanning procedure (scan time = 450 ms).*

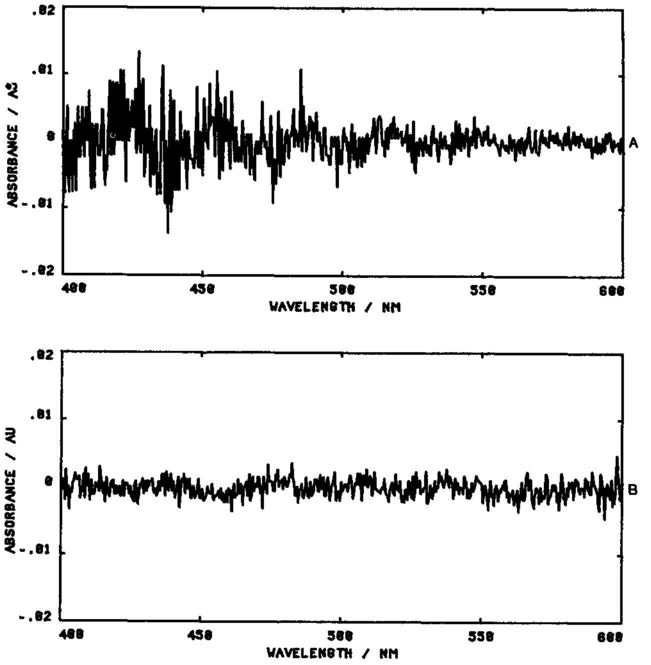

*Figure 3. Comparative absorbance base-line noise: (a) using the first scanning procedure (scan time = 63 ms); and (b) using the second scanning procedure (scan time = 450 ms).*

is dependent on the wavelength. The spectrum profile results from the fact that the sensors present sensitivities that decrease with the wavelength [14] in the visible region. Also, the halogen source employed shows a lower emission intensity in the blue-region of the spectra. This is a problem when the region of interest includes the range from 500 down to 400 nm. To correct for the various factors affecting the sensitivity in this region, software (the second scanning procedure) was developed to acquire spectral intensities employing variable integration time intervals that increase towards the lower end of the blue region. To do this the optical arrangement should project the red region of the spectrum on the first diodes, while the last diodes monitor the blue region. After adding this facility to the software, the intensity spectra of figure 2(*b*) was obtained. The scan time is, after doing this, no longer equal to the integration time. The integration times for the last diodes are greater than those for the first diodes. However, the data are more suitable for performing transmittance or absorbance calculations and allow the dynamic range of the electronics and the analogue-to-digital converter to be used optimally.

Procedures employing variable integration times have been reported in the literature [15–17]. However, these procedures have been carried out by collecting a number of spectra obtained at different scanning times. One final spectrum, which shows higher intensities without saturation, was recovered by joining parts of each scan together. These approaches are relatively time-consuming. For example, Wirz *et al.* [15] have obtained a complete spectrum (data acquisition and calculation) in approximately 60 s, and Lepla and Horlick [16] have obtained a spectrum in between 15 and 30 s. The procedure described here can optimize the dynamic range in only one scan with an elapsed time from 300 to 1850 ms.

Of course, the behaviour of the detector response to the emission spectrum in the wavelength range of interest should be known.

Figure 3 shows the base-line noise when both scanning procedures (figure 3(*a*)—first procedure; figure 3(*b*)—second procedure) were employed. These spectra were obtained under the same conditions as in figure 2. Figure 3(*a*) shows a higher noise in the 400 to 500 nm region: this is due to the low intensity signals obtained in this region when the first procedure is used. On the other hand, the variable integration time enhanced signal-to-noise ratio and the noise falls to the same magnitude over whole spectrum. All spectral data shown below were obtained employing the second scanning procedure to read the array.

The resolution obtained by instrument can be demonstrated by the emission spectra of a mercury source shown in figure 4(*a*). The estimated resolution is about 1·5 nm. However, when a smoothing procedure is applied to the data, this resolution decreases as shown in figures 4(*b*) and 4(*c*), which show the same spectrum after an unweighted sliding average smoothing of three and five photodiodes, respectively. When weighted smoothing of five diodes is employed, a spectrum similar to that shown in figure 4(*b*) is obtained.

Despite decreases in resolution, the smoothing procedure improves the signal-to-noise ratio. Table 1 shows the standard deviation of the absorbance measurements of the spectra shown in figures 3(*a*) and 3(*b*).

Sliding average smoothing employing five photodiodes gives the best result as far as noise is concerned. Figures 5(*a*), 5(*b*) and 5(*c*) show spectra from $5{\cdot}0 \times 10^{-2}$ mol.dm$^{-3}$ neodymium chloride without smoothing, and then smoothing with three and five diodes: noise is

*Table 1. Standard deviation in absorbance measurements.*

| | Original data | Three diodes average | Five diodes average |
|---|---|---|---|
| First scanning procedure | 0·0040 | 0·0024 | 0·0021 |
| Second scanning procedure | 0·0018 | 0·0013 | 0·0011 |

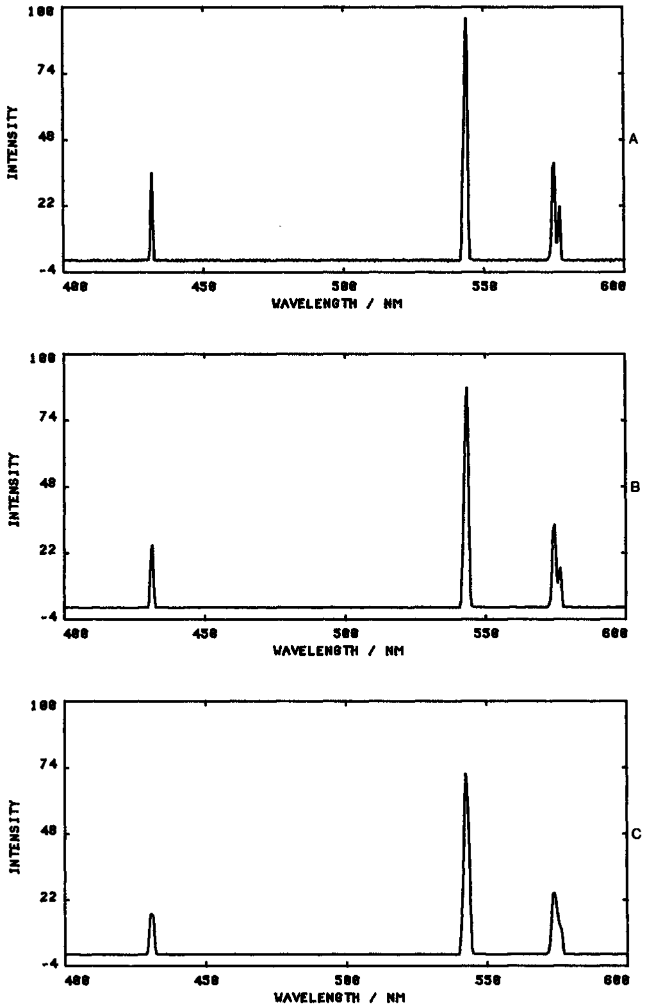

*Figure 4. Effect of smoothing procedures on the resolution: (a) original data; (b) unweighted sliding average of three diodes; and (c) unweighted sliding average of five diodes. Spectrum as shown in (a) can be used for calibration of the spectrophotometer.*

reduced as the smoothing procedure is applied. The following data result from the application of five diodes smoothing.

The precision of the absorbance measurement and the drift of the instrument were evaluated with the $1{\cdot}0 \times 10^{-4}$ mol.dm$^{-3}$ $KMnO_4$ test solution previously described; absorbance measurements were made at 525 nm. The result of averaging 18 spectra obtained in intervals of 1 s (1 s being the time between the last data transferred and the start of next scan) was 0·2029 ± 0·0013 AU. When 18 spectra scanned in intervals of 10 min were averaged, an absorbance of 0·2002 ± 0·0021 AU was obtained, which indicates that no significant drift exists.

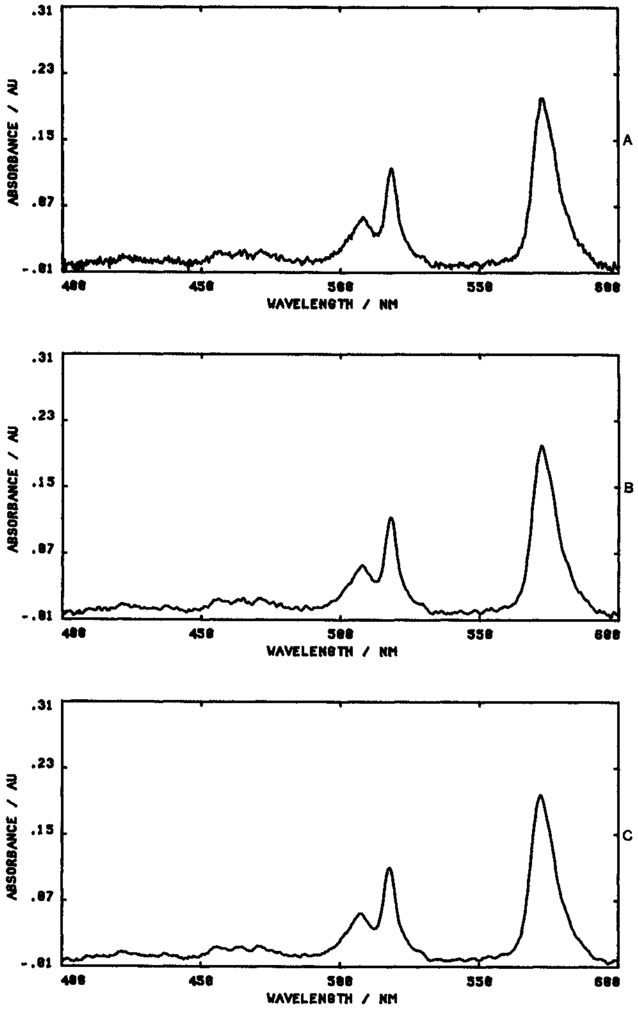

*Figure 5. Neodymium chloride solution absorbance showing the effect of the smoothing procedures: (a) original data; (b) unweighted sliding average of three diodes; and (c) unweighted sliding average of five diodes. The spectrum shown in (a) can be used for spectrophotometer calibration.*

The linearity was evaluated with solutions of $KMnO_4$ in the $5{\cdot}0 \times 10^{-5}$ to $8{\cdot}0 \times 10^{-4}$ mol.dm$^{-3}$ range. A straight line was observed up to $2{\cdot}5 \times 10^{-4}$ mol.dm$^{-3}$ ($r = 0{\cdot}99988$) with the first scanning procedure and up to $3{\cdot}5 \times 10^{-4}$ mol.dm$^{-3}$ ($r = 0{\cdot}99994$) with the second procedure. These results demonstrate the substantial improvements in the performance of the instrument when the variable integration time is employed.

### Acknowledgements

The authors are grateful to Dr R. E. Bruns for revisions to the first manuscript; to Mr G. C. Paixão for developing the plotter software; and to CNPq for financial support (process 403250/90-0).

### References

1. Agudo, M., Marcos, J., Ríos, A. and Valcárcel, M., *Analytica Chimica Acta*, **239** (1990), 211.

2. Brushwyler, K. R., Carter, L. D., and Hieftje, G. M., *Applied Spectroscopy*, **44** (1990), 1438.
3. Gerritsen, M. J. P., Kateman, G., van Opstal, M. A. J., Bennekom, W. P. and Vandeginste, B. G. M., *Analytica Chimica Acta*, **241** (1990), 23.
4. Poppi, R. J. and Pasquini, C., *Chemometrics and Intelligent Laboratory System*, **19** (1993), 243.
5. Melgarejo, A. G., Pavón, J. M. C. and Castro, A. R., *Analytica Chimica Acta*, **241** (1990), 153.
6. Lindberg, W., Clark, G. D., Hanna, C. P., Whitman, D. A., Christian, G. D. and Ruzicka, J., *Analytical Chemistry*, **62** (1990), 849.
7. León-Gonzáles, M. E., Pérez-Arribas, L. V., Santos-Delgado, M. J. and Polo-Díez, L. M., *Analytica Chimica Acta*, **258** (1992), 269.
8. Malcome-Lawes, D. J., Myers, J. and Pasquini, C., *Laboratory Microcomputer*, **7** (1988), 89.
9. Caceci, M. S., *Computers & Chemistry*, **13** (1989), 33.
10. Lepla, K. C. and Horlick, G., *Applied Spectroscopy*, **43** (1989), 1187.
11. Naffrechoux, E., Fachinger, C. and Suptil, J., *Analytica Chimica Acta*, **270** (1992), 187.
12. O'Haver, T. C., *Journal of Chemical Education*, **68** (1991), A147.
13. Poppi, R. J., Vazquez, P. A. M. and Pasquini, C., *Applied Spectroscopy*, **46** (1992), 1822.
14. EG&G Reticon, Product Description G-Series Solid State Line Scanners Data Sheet, EGG, Sunnyvale, California (1980).
15. Wirsz, D. F., Browne, R. J. and Blades, M. W., *Applied Spectroscopy*, **41** (1987), 1383.
16. Lepla, K. C. and Horlick, G., *Applied Spectroscopy*, **44** (1990), 1259.
17. Talmi, Y. and Simpson, R. W., *Applied Optics*, **19** (1980), 1401.

